# Penicillin allergy de‐labelling in the critical care unit: Simulations to design an intravenous drug provocation test that mirrors the plasma profile of an enteral challenge

**DOI:** 10.1002/bcp.70388

**Published:** 2025-12-02

**Authors:** Cleodie Swire, Sean Keane, Reya Vinay Shah, Toqa El‐Nahhas, Iao Lei, Salma Alamin, Niall Conlon, Dagan Osborne Lonsdale

**Affiliations:** ^1^ Department of Clinical Pharmacology & Therapeutics St. George's University Hospitals Foundation NHS Trust London UK; ^2^ School of Health and Medical Sciences St George's University of London London UK; ^3^ Trinity College Dublin Dublin Ireland; ^4^ Department of Anaesthesiology Intensive Care and Pain Medicine, St James's Hospital Dublin Dublin Ireland; ^5^ Department of Critical Care St George's University Hospitals NHS Foundation Trust London UK; ^6^ Department of Immunology St James's Hospital Dublin Dublin Ireland

**Keywords:** antibiotics, drug allergy, intensive care, pharmacokinetics

## Abstract

The majority of penicillin allergy labels are incorrect and a label of penicillin allergy is associated with worse outcomes and increased use of healthcare services. Penicillin allergy assessment and de‐labelling may be considered in critically ill patients. An oral penicillin challenge is a common component of most penicillin allergy protocols. In critical illness, the enteral route may not be an option and is less reliable due to altered absorption kinetics. In this study, simulations were undertaken to determine an intravenous infusion dosing schedule for critically ill patients that matches the concentration–time profile of an oral penicillin drug provocation test in the general population. These simulations may help clinicians develop procedures for intravenous infusion penicillin drug provocation tests for critically ill patients.

**What is already known about this subject?**

- The label of penicillin allergy is incorrect in 80%–90% of patients, and a label of penicillin allergy is associated with worse outcomes and increased use of healthcare services.
- De‐labelling services are successful in removing incorrect penicillin allergy labels. Sometimes, this is done with the use of a drug provocation test where an oral dose of penicillin is given and the patient observed over a certain period of time for the development of a reaction.
- In critical illness, pharmacokinetics are altered, and enteral administration of drugs is often unreliable or impossible.

**What this study adds?**

- Absorption of enterally administered amoxicillin in critically unwell patients is likely to lead to reduced exposure during a drug provocation test compared to the exposure in non‐critically ill patients. The impact of this for drug provocation tests is unknown.
- We present a protocol for an intravenous infusion penicillin drug provocation test that mirrors the plasma profile of an enteral challenge. This protocol could be considered as part of comprehensive penicillin allergy de‐labelling protocols in critically unwell patients.

## 1 INTRODUCTION

The impact of an incorrect penicillin allergy label is a challenge in critical care. The label of a penicillin allergy is associated with worse healthcare outcomes for the individual,^1^ including an increase in acquisition of drug resistant infections^2^ and exposure to antibiotics with a less favourable adverse effect profile compared to β‐lactams, including quinolones.

Discussions around penicillin allergy are common, with 6%–10% of patients reporting penicillin hypersensitivity.^1^, ^3^ The incidence of true penicillin allergy is likely to be far lower, with 5%–20% of people who report an allergy actually having one.^3^, ^4^, ^5^ Assessment of allergy history leads to over 60% patients being labelled as low risk. Patients are stratified as low risk if they report symptoms that are consistent with minor non‐immunological side effects or other non‐allergic phenomena.^6^ This group is suitable for direct drug provocation test without skin sensitization testing.^6^ Identifying those who *do not* have a penicillin allergy through assessment and testing where appropriate (allergy de‐labelling) is a potential strategy to optimize infection management and antimicrobial stewardship in critical care. Studies evaluating enteral penicillin provocation tests in critical care have demonstrated these to be safe in individuals assessed to be at low risk of true penicillin allergy, and this practice has been embedded into routine care in some hospitals.^7^, ^8^, ^9^ Amoxicillin is a commonly used agent for this purpose via the enteral route.

For some, including critically ill patients, the enteral route of drug administration may be an imperfect choice. In this manuscript, the term ‘critically ill patients’ refers to individuals admitted to the intensive care unit, which reflects the cohorts in the pharmacokinetic and allergy publications that this study is based on. Reasons for admission vary in practice but typically are physiological instability secondary to medical or surgical conditions and a requirement for interventions such as invasive haemodynamic monitoring, vasopressor support, mechanical ventilation (invasive or non‐invasive) or renal replacement therapy. Absorption kinetics are highly variable in critical illness through mechanisms such as altered haemodynamics and ileus.^10^, ^11^ Some pathologies and clinical situations, which are common in critical illness, will render the enteral route of administration unavailable. Forsberg et al undertook a systematic review of oral drug absorption in critical illness. They found no published data on penicillin absorption in this patient group, and in the small number of antibiotics that do have data, absorption was lower compared to non‐critically ill populations.^12^

For drugs that are more well studied, the impact of critical illness on absorption kinetics is significant. For example, Heyland et al undertook a study on the impact of critical illness on paracetamol kinetics.^13^ In critically ill populations, they found a 55% reduction in maximum concentration ( $C_{\max}$) and 20% reduction in the area under the concentration–time curve compared to healthy volunteer controls. The time to maximum concentration ( $T_{\max}$) was 3.5 times (75 min) longer in critically ill patients compared with healthy individuals. Similar results were found by Tarling et al.^14^

A protocol for an IV penicillin infusion drug provocation test was recently published by Molina‐Molina et al, with accompanying safety data from their experience over a retrospective 4‐year period.^15^ The patient population in the Molina‐Molina et al study were general outpatients and inpatients. To our knowledge, there is no similarly published protocol for critically ill patients.

In this report, we present a simulated pharmacokinetic study of a protocol for an IV amoxicillin drug provocation test in critical illness. We have taken published parameters from pharmacokinetic models of amoxicillin and aim to demonstrate the feasibility of providing a pharmacokinetic profile through IV infusion that mirrors that of the clinical dose used in an oral amoxicillin drug provocation test.

## 2 METHODS

### 2.1 Pharmacokinetic models

A literature search was conducted to identify studies with relevant pharmacokinetic models for amoxicillin upon which to base simulations for this work. As amoxicillin has dose‐dependent absorption kinetics, studies for enteral doses other than 500 mg were excluded. There are no universally accepted criteria for choosing one pharmacokinetic model over another and no pharmacokinetic study comparing oral and IV amoxicillin kinetics in a critically ill population at doses used for drug provocation tests. Model selection was therefore based on size of sample and a qualitative review of published model fit diagnostics.

Simulations were undertaken in R using the rxode2 package.^16^, ^17^ The code file is provided as Supporting Information.

Three scenarios were simulated, fixing weight (the only covariate) at 70 kg:

- A reference model of enteral administration in the non‐critically ill population.
- A simulated enteral model in critically ill patients to illustrate the potential impact of critical illness on absorption kinetics.
- A simulated IV model in critically ill patients using a similar infusion rate strategy to that suggested by Molina‐Molina et al and amoxicillin pharmacokinetic parameters from Lonsdale et al.

10 000 patients were simulated for each scenario. Concentrations were generated every 7.5 min for each simulated patient, between 0 and 12 h after dosing.

### 2.2 Enteral amoxicillin model in non‐critically ill patients

A two‐compartment oral‐absorption model with Michaelis–Menten and Savic transit compartment absorption kinetics was simulated. Fixed and random effects parameters were taken from de Velde et al.^18^

Amoxicillin dose was 500 mg. This was selected because it is the established standard for drug provocation testing, particularly when the index agent is amoxicillin or an unknown penicillin. This approach aligns with the British Society for Allergy and Clinical Immunology guideline,^6^ which outlines risk stratification and standardized drug provocation testing protocols for non‐allergists.

### 2.3 Enteral amoxicillin model in critically ill patients

A two‐compartment oral absorption model with Michaelis–Menten and Savic transit compartment absorption kinetics was simulated. Absorption parameters were taken from de Velde et al^18^ and distribution and elimination parameters from Lonsdale et al.^19^ To simulate the effect of critical illness on absorption, we increased mean transit time and reduced bioavailability and Vmax to provide a similar impact on kinetics to that found by Heyland et al in their paracetamol study.^13^ Amoxicillin dose was 500 mg.

### 2.4 IV infusion model

A two‐compartment IV model was simulated. Fixed and random effects parameters were taken from Lonsdale et al.^19^ The IV infusion utilized incremental increases in rate, adapted from a protocol published by Molina‐Molina et al.^15^ The 1% of the dose was delivered over the first 10 min, 9% over the next 20 min, and the remainder over an hour (total infusion time 90 min). Stability data are in keeping with this being an acceptable duration of time for amoxicillin to be left.^20^ A range of doses were tested in 50 mg increments, and we present results of a simulated total amoxicillin dose of 200 mg in this report as this resulted in the most similar median maximum concentration ( $C_{\max}$) to the enteral model in non‐critically ill patients.

### 2.5 Analysis of simulations

End points were maximum concentration ( $C_{\max}$), time to maximum concentration ( $T_{\max}$) and exposure (quantified as area under the time–concentration curve, $\mathrm{AU}C_{0-\infty }$). Parameters are presented as median with 5th and 95th centile range. $\mathrm{AU}C_{0-\infty }$ was calculated using simulated clearance:

$$\mathrm{AU}C_{0-\infty }=\frac{\text{simulated bioavailability}\;x\;\text{dose}}{\text{simulated clearance}}$$

## 3 RESULTS

Published two‐compartment pharmacokinetic models of amoxicillin from de Velde et al^18^ and Lonsdale et al^19^ were chosen for simulation. Our view was that these provided a robust set of models upon which to base our simulations. The de Velde et al study selected for the non‐critically ill population is the largest study (28 people) identified after a 500 mg oral dose of amoxicillin; sources not chosen include Spyker et al (24 people)^21^ and Arancibia et al (nine people).^22^ The Lonsdale et al study selected to provide the parameters in critical illness is the largest study (49 adults, 24 children and seven neonates) with published pharmacokinetic parameters for amoxicillin for this population; sources not chosen include Carlier et al (13 people)^23^ and Mimoz et al (12 people).^24^

**FIGURE 1:**
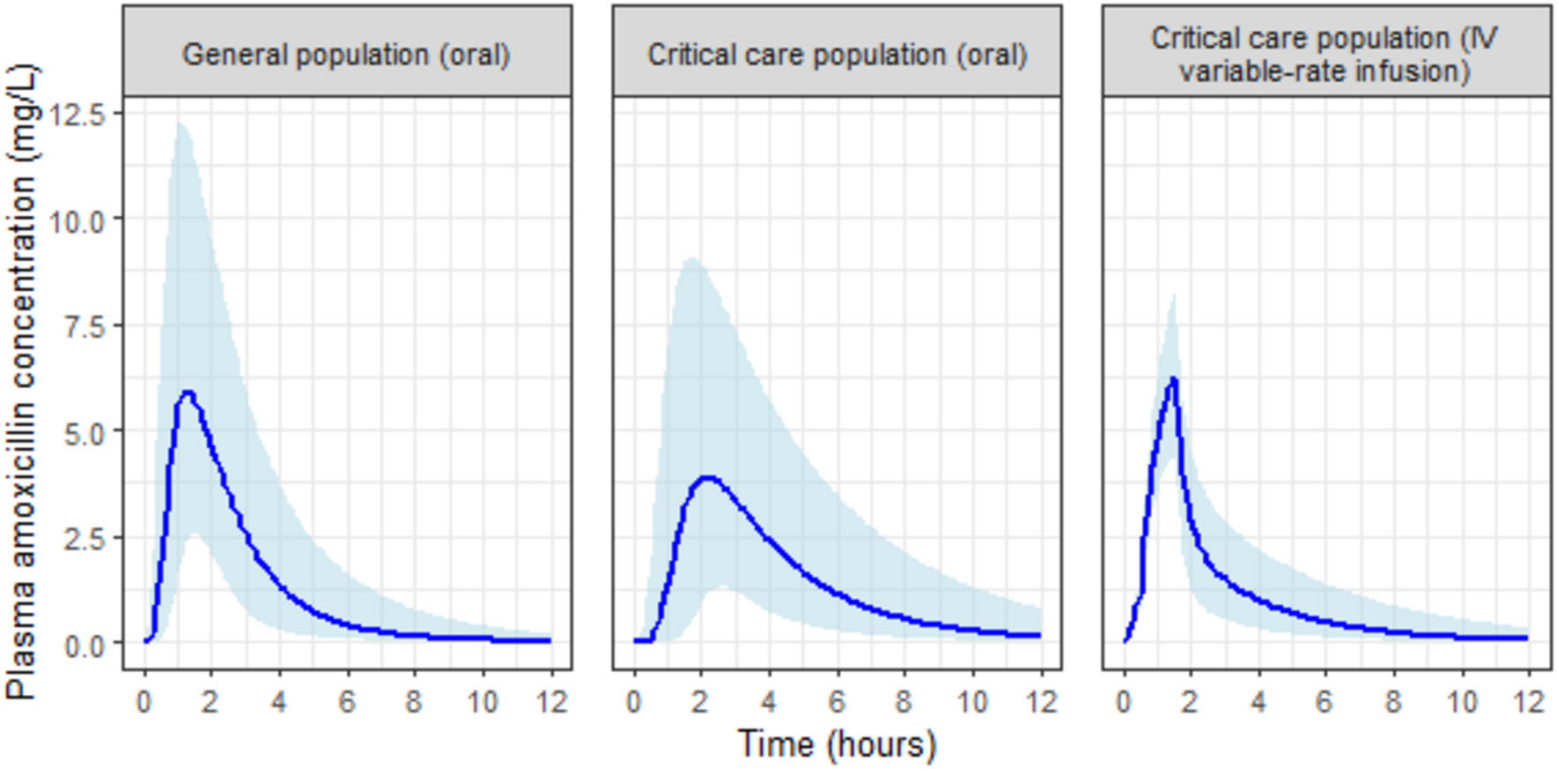
Concentration–time plots from three simulated scenarios of a single dose of amoxicillin. Amoxicillin dose is 500 mg for oral scenarios and 200 mg for the IV variable‐rate infusion. Solid line represents median concentration at each time point, and shaded area the 5th–95th centiles.

Simulations of a 200 mg variable rate infusion of amoxicillin in a critically ill population achieved a similar pharmacokinetic profile to a 500 mg enteral dose in a non‐critically ill population (Table 1). Median $C_{\max}$ was 6.27 mg/L, and $T_{\max}$ was 1.5 h in the simulated IV scenario compared to 6.65 mg/L and 1.25 h from the oral route in the non‐critically ill. Concentration–time profiles from the three simulated scenarios are presented in Figure 1.

**TABLE 1:** Summary pharmacokinetics from three simulated amoxicillin experiments. $C_{\max}$ is maximum concentration, $T_{\max}$ is time from administration to maximum concentration, AUC is area under the concentration–time curve for a single dose.

Pharmacokinetic parameter	Simulated scenario		
	General population (oral) *N* = 10 000^a^	Critical care population (oral) *N* = 10 000^a^	Critical care population (IV variable‐rate infusion) *N* = 10 000^a^
*C*_*max*_ (mg/L)	6.65 (3.10, 13.38)	4.86 (2.11, 10.67)	6.27 (4.38, 8.26)
*T*_*max*_ (hours)	1.25 (0.75, 2.25)	2.25 (1.25, 4.13)	1.50 (1.50, 1.50)
AUC (mg.h/L)	16 (8, 33)	18 (8, 41)	13 (7, 24)

^a^ Median (5% centile, 95% centile).

## 4 DISCUSSION

Our simulations predict that a 200 mg infusion of amoxicillin given to critically ill populations with staged infusion rates will confer a similar time–concentration profile to that expected in a healthy population given an oral 500 mg dose of amoxicillin. This, alongside studies of IV penicillin drug provocation tests in healthy patients, may provide clinicians with some assurance when considering protocols for IV amoxicillin drug provocation test in the intensive care unit. Similarly, we offer some evidence of the lower exposure expected through the use of enteral administration in this population.

Recent hypotheses based on the law of mass action have argued that adverse drug reactions are all dose dependent and explanations for situations where this was not felt to be the case have been proposed.^25^ Based on this, it is expected that increased $C_{\max}$ may lead to a higher rate of harm. For this reason, this study aimed to match the $C_{\max}$ of doses used in established enteral drug provocation test protocols.

Pharmacokinetic model‐informed doses are not (to our knowledge) used in existing penicillin allergy de‐labelling protocols. Molina‐Molina et al used a standard licensed dose of 1000 mg of amoxicillin.^15^ Immediate reactions in this study were higher in the group administered an IV provocation challenge, although interpreting this result is challenging as the study was not randomized, and the intravenous group had a higher risk of a true allergy at baseline and were preferentially chosen for the IV group for safety reasons.

In our view, the IV route of administration in critically ill patients has a number of advantages over enteral. Principally, this is in the reliable achievement of meaningful exposure that may be lost in the setting of unreliable and lower absorption kinetics found in critical illness. Further, in the event of a reaction to the drug, the IV infusion can be stopped immediately, reducing exposure to the drug (an option not available once an oral dose is administered or with an IV bolus).

We acknowledge the ethical considerations involved in performing penicillin allergy assessment and testing in critically ill patients. Spurious penicillin allergy labels are associated with increased morbidity and increased mortality in both inpatient and outpatient cohorts.^1^, ^26^ In a detailed discussion of the ethics of antibiotic allergy, Xiang et al conclude that prescription of first‐line antibiotics to patients carrying an allergy label is often ethically appropriate.^27^ The authors also highlight the requirement for more widespread testing. The safety and feasibility of non‐allergist led allergy assessment and testing in critically ill patients have been assessed in a prospective study,^9^ with no adverse events reported. In patients assessed as having a low‐risk allergy label, the risk–benefit profile often favours testing over deferral. Testing is not recommended for patients assessed to be at high risk for allergy, and decisions should always be individualized, ideally involving the patient or their nominated decision‐maker. The overarching aim is to avoid denying critically ill patients the benefits of optimal antimicrobial therapy due to inaccurate historical allergy labels.

Clinicians may be concerned that the IV route may present a large and rapid drug burden at the site of administration. The IV route is associated with a risk of rapidly evolving reactions, but it is suggested that the IV route, using incremental doses, is easier to control, as symptoms are likely to occur earlier during IV than oral administration and may appear after a smaller dose.

Further mitigation may be found from a large administration volume (with rate adjusted to deliver dose), or central administration, where the impact of cardiac output is likely to dilute drug administered at this rate to a concentration commensurate with that found in the portal venous system after an oral dose. Infusion rates for a range of reconstitution volumes are provided in Table 2.

**TABLE 2:** Stepwise infusion rates for a 200 mg intravenous amoxicillin dose. A range of potential infusion volumes are shown.

Infusion volume	Rate of amoxicillin infusion (mL per min)		
	50 mL (4 mg/mL)	250 mL (0.8 mg/mL)	500 mL (0.4 mg/mL)
Period 1: 10 min infusion (1% of total dose)	0.05	0.25	0.50
Period 2: 20 min infusion (9% of total dose)	0.23	1.13	2.25
Period 3: 60 min infusion (90% of total dose)	0.75	3.75	7.50

*Note*: This does not take into account line volume (e.g. if central line used).

Small dose increments with no intervals can potentially desensitize and blunt hypersensitivity reactions. We feel this is unlikely to occur with our proposed protocol as it is markedly different to desensitization protocols. The starting rate of our modelled infusion is 12 mg/h compared to 0.006 mg/h for a β‐lactam desensitization protocol^28^; our infusion duration is also shorter (90 min compared to 3 h).

Guidelines on penicillin allergy assessment and testing in the general population advocate for drug provocation tests without the use of skin sensitization testing for patients assessed to be low risk based on their allergy history.^6^ Oral drug provocation tests have been shown to have an excellent safety profile in critical care populations.^7^, ^8^ We propose that IV drug provocation tests could also be considered for low‐risk penicillin allergy in critically ill patients. This would allow more patients to benefit from allergy de‐labelling as skin sensitization testing, typically led by immunology or allergy specialist services, is not readily available at many centres.

Our work shares the same limitations of other simulation based science, in that it is dependent on the models used and the precision of the parameter estimates within. The chosen models were selected largely on the basis of expert opinion. Our work is particularly limited by the absence of pharmacokinetic data from oral administration in the critically ill population. However, we do not believe a study to accurately determine the impact of critical illness on absorption kinetics of amoxicillin is likely to be undertaken. The drug is seldom used through this route and such a study would likely require a relatively large sample size to understand the variability in this population. Our model of IV infusion is more robust as it is based on a relatively large pharmacokinetic study. In our view, if a clinical study is felt necessary to confirm our simulations, the focus should be on confirming the pharmacometrics and safety of the IV infusion approach.

Verification of an individual's allergy status should form a key part of optimizing their care but may be limited in critical illness by the unreliability of the enteral route and access to specialist allergy services. This study used pharmacokinetic modelling to inform suggested IV infusion rates for critically ill patients, which approximate the concentration–time profile of the amoxicillin dose used in penicillin drug provocation tests in the general population. This could be used to add the option of an IV route to allergy testing protocols and improve access to the benefits of penicillin allergy de‐labelling.

## AUTHOR CONTRIBUTIONS

Cleodie Swire, Sean Keane and Dagan Osborne Lonsdale conceptualised the study. Cleodie Swire and Dagan Osborne Lonsdale designed the pharmacokinetic simulation methodology, developed the R code, performed the pharmacokinetic simulations, and drafted the original manuscript. Reya Vinay Shah reviewed and advised on the R code. Toqa El‐Nahhas and Iao Lei assessed the practical feasibility of the proposed clinical application. Salma Alamin and Niall Conlon provided guidance on the proposed clinical application and ethics from an immunological perspective. All authors reviewed the manuscript, contributed to revisions, and approved the final version for submission.

## CONFLICT OF INTEREST STATEMENT

None of the authors disclose any conflict of interest directly relevant or directly related to this work.

## Supporting information


**Data S1.** Supporting Information.

## Data Availability

R markdown file available in the Supporting Information of this article.
